# Spontaneous bladder rupture induced pseudo-acute kidney injury: A case report and literature review

**DOI:** 10.1097/MD.0000000000045558

**Published:** 2025-10-31

**Authors:** Tianle Yu, Chunyu Cai, Jia Shi, Yu Ji Jiang

**Affiliations:** a Department of Nephrology, Affiliated Hospital of Yanbian University, Yanji, China.

**Keywords:** pseudo-renal failure, retrograde cystography, spontaneous rupture of the urinary bladder (SRUB), Urinary ascites

## Abstract

**Rationale:**

Spontaneous rupture of the urinary bladder (SRUB) is a rare, easily missed cause of urinary ascites that mimics intrinsic renal failure through peritoneal reabsorption of urinary solutes (“reverse peritoneal dialysis”). Rapid recognition prevents unnecessary dialysis and life-threatening complications. This case underscores key diagnostic pointers (especially the ascites/serum creatinine (SCr) ratio and the decisive role of computed tomography [CT] cystography).

**Patient concerns:**

A previously healthy 45-year-old man presented 5 days after heavy alcohol intake with severe lower-abdominal pain, progressive abdominal distension, oliguria (~300 mL/24 hours), and dyspnea. Initial tests showed SCr 7.6 mg/dL, blood urea nitrogen 108 mg/dL, sodium 125 mmol/L, and potassium 6.0 mmol/L.

**Diagnoses:**

Ascitic fluid analysis revealed creatinine 131 mg/dL with an ascites/SCr ratio of ~17:1, indicating urinary ascites. Retrograde CT cystography demonstrated intraperitoneal contrast extravasation from the right bladder wall, confirming SRUB with pseudo-acute kidney injury.

**Interventions:**

Initial hemodialysis and large-volume paracentesis (2.0–2.5 L/day) did not improve distension. A Foley catheter was placed, CT cystography was performed, and the patient underwent surgical bladder repair with urinary ascites evacuation and postoperative bladder drainage.

**Outcomes:**

Urine output increased promptly. Electrolytes and SCr normalized within 48 hours after repair. A day-7 cystogram showed no leak; the catheter was removed at 3 weeks. At 3-month follow-up, there was no recurrence.

**Lessons:**

Consider SRUB in patients with unexplained ascites, oliguria, and acute azotemia (particularly after alcohol binge drinking). Measuring the ascites/SCr ratio (>2) provides powerful evidence of urinary ascites, and CT cystography is the preoperative diagnostic gold standard. Early definitive repair reverses electrolyte derangements rapidly and avoids unnecessary renal replacement therapy and potential fatal peritonitis.

## 1. Introduction

Spontaneous bladder rupture (SBR) is a rare yet life-threatening condition that typically arises from a weakened bladder wall or increased intra-abdominal pressure.^[1]^ Urinary ascites, resulting from the intraperitoneal leakage of urine, may present similarly to other causes of acute abdomen. The absorption of urine through the peritoneum can lead to electrolyte disturbances (including hyperkalemia, hyponatremia, and metabolic acidosis) manifesting as pseudo-renal failure (PRF).^[2]^ A delayed diagnosis may result in severe complications or even death. This case analysis examines the pathophysiological mechanisms, diagnostic approaches, and treatment strategies for PRF and urinary ascites resulting from SBR. Imaging techniques, particularly computed tomography (CT) cystography, are crucial for definitive diagnosis, while the ascitic fluid/serum creatinine (SCr) ratio serves as a key diagnostic marker. Furthermore, the SCr/cystatin C (CysC) ratio (>2 L/dL) may serve as a clue for the clinical diagnosis of pseudo-acute kidney injury (pseudo-AKI) engendered by urinary ascites. This case underscores the importance of early recognition to prevent unnecessary renal replacement therapy.

## 2. Case report

This retrospective case analysis was approved by the institutional ethics committee.

### 2.1. Patient information

A 45-year-old man presented to the nephrology department on November 10, 2024, with a 5-day history of oliguria, abdominal pain, abdominal distension, and elevated SCr levels. The patient described that after consuming approximately 300 mL of 52% alcohol on the evening of November 5, he developed abdominal pain and bloating the following morning. He reported difficulty in urination, passing only scant amounts of dark-colored urine (approximately 300 mL in total over the subsequent 24 hours) suggestive of acute urinary retention. The patient reported difficulty in urination with a weakened urinary stream, abdominal distension, and suprapubic discomfort, consistent with transient urinary outflow obstruction. Initial laboratory tests conducted at our hospital revealed the following: SCr 7.6 mg/dL, blood urea nitrogen (BUN) 38.7 mmol/L, potassium 6.0 mmol/L, sodium 125 mmol/L, and chloride 80 mmol/L. He was provisionally diagnosed with acute renal failure and was admitted for further evaluation.

### 2.2. Past medical history

The patient had no significant medical history. He denied a history of hypertension, diabetes, or infectious diseases such as tuberculosis. There was no family history of genetic disorders.

### 2.3. Physical examination

Upon admission, the patient’s vital signs were recorded as follows: temperature 36.1°C, pulse 91 beats per minute, respiratory rate 17 breaths per minute, blood pressure 149/96 mm Hg, and oxygen saturation at 100%. The patient appeared visibly distressed, complaining of severe lower-abdominal pain and progressive abdominal distension. Abdominal examination revealed marked distension, with a measured circumference of 90 cm. The abdominal wall was mildly tense and exhibited diffuse tenderness with localized rebound pain. Shifting dullness was positive on percussion, consistent with significant ascites.

### 2.4. Laboratory investigations

- Complete blood count: white blood cells 26.09 × 10^9^/L; neutrophils 88.4%; hemoglobin 167 g/L; platelets 251 × 10^9^/L; C-reactive protein 109.20 mg/L.
- Urinalysis: leukocytes + 3; urinary microalbumin > 0.1; urine albumin + 2; glucose + 1; occult blood + 4.
- Biochemistry: procalcitonin 1.36 µg/L; creatine kinase 1009 U/L; total bilirubin 54.60 µmol/L; total protein 82 g/L; albumin 49 g/L; alanine aminotransferase 96 U/L; aspartate aminotransferase 106 U/L; lactate dehydrogenase 479 U/L; SCr 7.64 mg/dL; BUN 108 mg/dL; CysC 1.18 mg/L; sodium 125 mmol/L; potassium 6.0 mmol/L; chloride 80 mmol/L; amylase 83 U/L; fasting plasma glucose 7.2 mmol/L; random plasma glucose 14.2 mmol/L.
- Immunology: complement C3 0.95 g/L; negative for antinuclear antibody, anti-double-stranded DNA antibody, antineutrophil cytoplasmic antibody, and anti-glomerular basement membrane antibody.

### 2.5. Imaging studies

**Figure 1.**
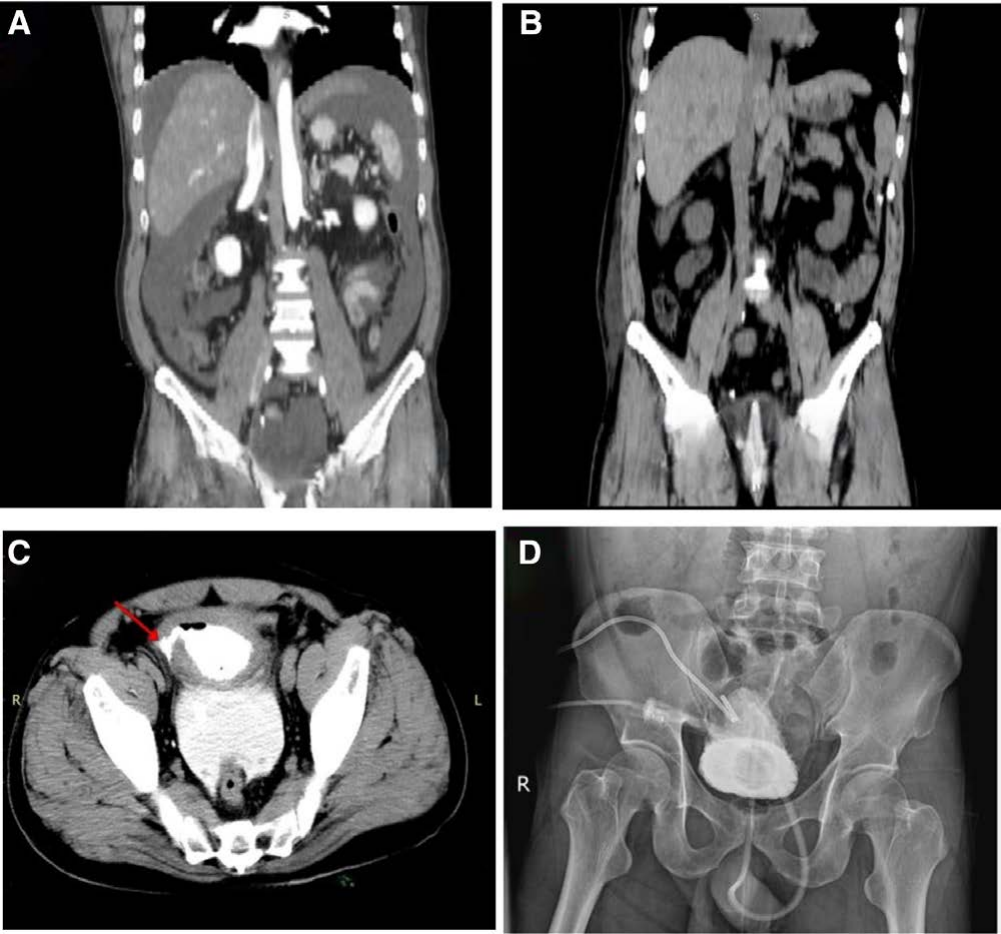
CT imaging of spontaneous bladder rupture. (A) Admission non-contrast abdominal CT demonstrating large-volume ascites. (B) Follow-up abdominal CT 3 days after bladder repair and catheterization showing near-complete resolution of ascites. (C) Abdominal CT after retrograde cystography with intraperitoneal contrast extravasation (arrow) and intravesical gas. (D) Fluoroscopic image during retrograde cystography confirming contrast leak.

- Abdominal CT (non-contrast): fatty liver, gallstones, suspected cholestasis, possible left adrenal hyperplasia; bladder wall thickening, prostatic calcification but no abnormalities in prostate size or morphology; diffuse thickening of the small intestine and large-volume abdominopelvic effusion.
- Contrast-enhanced abdominal CT: diffuse thickening of the small intestine and ascending colon walls; irregularity of the right bladder wall; findings suggest peritonitis and large-volume ascites (Fig. 1).

### 2.6. Clinical course and outcome

After admission, daily urine output remained < 300 mL, with progressive abdominal distension and worsening dyspnea. Hemodialysis was initiated for acute renal failure, and daily paracentesis drained 2000 to 2500 mL of ascitic fluid, though abdominal girth showed no significant reduction.

Initial ascitic fluid analysis: yellow and slightly turbid; Rivalta test negative; glucose 7.25 mmol/L; total protein 5 g/L; lactate dehydrogenase 167 U/L; total cell count 770 cells/µL; sterile culture; no malignant cells on exfoliative cytology. The cause of ascites remained unclear. Further testing revealed an ascitic fluid creatinine level as high as 131 mg/dL, indicating urinary ascites.

A Foley catheter was placed, and retrograde cystography revealed extravasation of contrast from the right bladder wall into the abdominal cavity, with rapid accumulation of high-density fluid, confirming intraperitoneal bladder rupture.

**Figure 2.**
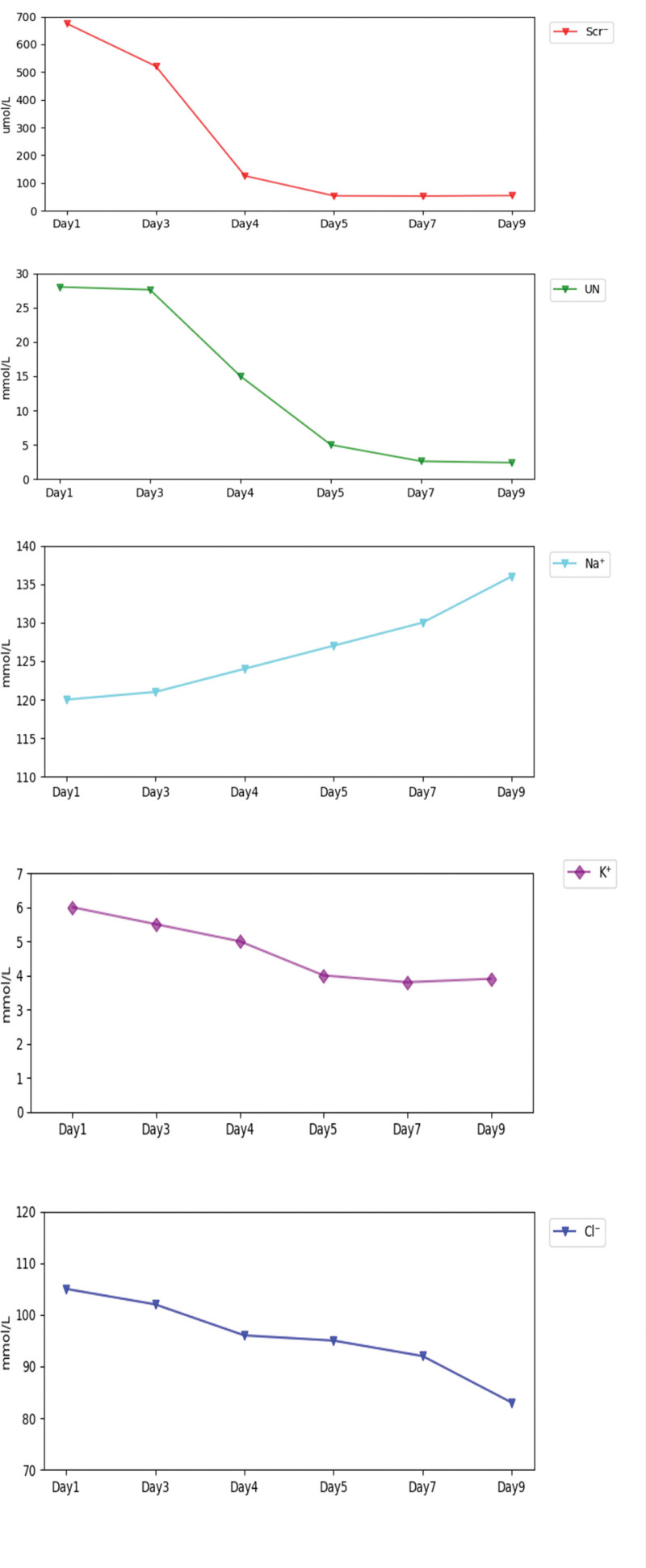
Trends in serum biochemical markers during the first 9 days of hospitalization, including blood urea nitrogen, creatinine, sodium, potassium, and chloride levels. All parameters gradually returned to normal following laparoscopic bladder repair and 48 hours of indwelling catheterization.

The patient was transferred to urology for bladder repair. Intraoperative findings confirmed bladder rupture and the presence of urinary ascites, which was evacuated. Serum BUN and SCr returned to normal within 48 hours postoperatively. Notably, refractory hyponatremia and hypochloremia on admission, which persisted despite hypertonic saline supplementation, also resolved rapidly following surgical repair (Fig. 2).

## 3. Discussion

Although SBR is relatively rare, it can be life-threatening. When urine leaks into the peritoneal cavity, it may lead to urinary ascites. Due to the high concentrations of creatinine and nitrogenous waste products in urine, absorption through the peritoneum induces a “reverse peritoneal dialysis” effect, resulting in hyponatremia, hyperkalemia, elevated SCr, and azotemia, which may present as PRF.^[3]^ This pathological process mimics reverse peritoneal dialysis, exacerbating electrolyte disturbances and misleading renal function markers. Such abnormalities can worsen symptoms and heighten the risk of misdiagnosis, often resulting in unnecessary renal replacement therapy.^[4]^ Early diagnosis and prompt intervention are crucial. In certain cases, bladder healing can be achieved through catheterization and ascitic drainage alone, with both BUN and SCr typically returning to normal within ~24 hours. Therefore, timely recognition and treatment of SBR and its complications are essential to prevent overtreatment and improve patient outcomes.

Urinary ascites can arise from non-traumatic bladder injuries or neurogenic conditions, including congenital spinal anomalies, spinal cord injuries, or multiple sclerosis. It may also be associated with excessive alcohol consumption and subsequent minor trauma.^[5]^ In patients presenting with unexplained massive ascites, it is essential to test the creatinine levels in the ascitic fluid. Typically, the ascites creatinine/SCr ratio > 1 is suggestive of urinary ascites, and a ratio > 2 provides stronger diagnostic evidence. In the present case, the patient was misdiagnosed with acute renal failure and underwent dialysis, which not only failed to alleviate symptoms but also exacerbated the condition. Although post-renal failure due to spontaneous rupture of the urinary bladder (SRUB) initially does not involve intrinsic renal damage, a delayed diagnosis can result in AKI, peritonitis, septic shock, and severe electrolyte imbalances such as hyponatremia and hyperkalemia.^[6]^ These potentially life-threatening complications align with the findings in our case, thereby emphasizing the diagnostic challenges posed by pseudo-AKI in clinical practice. Without prompt recognition, the condition may deteriorate and become fatal.

Bladder rupture is most frequently associated with abdominal or pelvic trauma, but it can also occur spontaneously or as a result of medical intervention, such as postsurgical or endoscopic procedures. Ruptures may be classified as intraperitoneal or, more commonly, extraperitoneal.^[7]^ SRUB refers to bladder perforation occurring without external trauma or direct force. Previous estimates suggested an incidence rate of 1 in 126,000; however, recent data indicate a rate of approximately 1 in 50,000, with a mortality rate ranging from 47% to 80%, primarily due to undiagnosed underlying causes.^[4]^ Alcohol abuse is frequently identified as a significant precipitating factor for SRUB. This may be attributed to several mechanisms: alcohol-induced diuresis; exacerbation of preexisting benign prostatic hyperplasia, resulting in bladder outlet obstruction; alcohol-related nausea and vomiting, which increase intra-abdominal pressure; sensory impairment during intoxication, leading to bladder over-distension; and unnoticed or forgotten minor trauma.^[8]^ A review of 713 SRUB cases identified alcohol abuse as the leading cause, accounting for 39.27% of cases. Importantly, nearly 75% of reported cases were initially misdiagnosed.^[9]^ In our case, the patient was initially misdiagnosed with incomplete intestinal obstruction following recent heavy alcohol consumption. This underscores the need for heightened clinical suspicion of SRUB in patients with urinary ascites, acute elevation of SCr, oliguria, and signs of peritonitis, especially in the context of alcohol use.

Extraperitoneal bladder rupture is commonly associated with pelvic fractures and typically presents with abdominal pain and dysuria. In contrast, intraperitoneal rupture is characterized by a classic triad of gross hematuria, abdominal pain, and urinary difficulty. However, clinical features can be nonspecific, including anuria with intraperitoneal free fluid, abdominal distension, and oliguria.^[9]^

Diagnosing SRUB is challenging due to the absence of trauma history and the nonspecific nature of symptoms. Key diagnostic clues include: sudden and persistent lower abdominal pain, often accompanied by nausea, vomiting, dysuria, oliguria, or hematuria; physical findings of peritoneal irritation and shifting dullness; paracentesis typically revealing pale yellow or pink fluid. SRUB should be considered in patients presenting with unexplained ascites. Laboratory findings may show abnormalities in blood counts, renal and liver function, and myocardial enzymes. Peritoneal absorption of urinary solutes can cause elevations in BUN and SCr, mimicking acute renal failure. An ascites creatinine/SCr ratio > 1.0 is considered suggestive of urinary ascites, while a ratio > 2.0 provides stronger diagnostic evidence. Ultrasound is the first-line imaging modality and may reveal poor bladder filling, perivesical hematoma, or ascites. Non-contrast CT has limited sensitivity but may help exclude other etiologies. CT cystography is the gold standard for preoperative diagnosis. Additionally, methylene blue instillation via a Foley catheter, followed by observation of ascitic fluid discoloration, offers a simple and reliable bedside diagnostic method.^[10–12]^

Creatinine, a product of muscle metabolism, is excreted primarily via glomerular filtration and is commonly used to estimate the glomerular filtration rate. CysC is a low-molecular-weight protein secreted by nucleated cells, freely filtered by glomeruli and almost completely reabsorbed and catabolized by renal tubules. Its serum concentration is less influenced by extrarenal factors and provides a more accurate reflection of glomerular filtration rate.^[13]^ The SCr/CysC ratio can reduce confounding and improve sensitivity for early detection of renal impairment. In patients with urinary ascites, SCr is often elevated while CysC remains stable. A SCr/CysC ratio > 2 (L/dL) on 3 consecutive measurements may indicate the potential for pseudo-AKI.^[10]^ Following catheterization and ascitic drainage, both creatinine levels and the ratio typically normalize. Although this ratio has diagnostic potential, surgical exploration remains the gold standard for confirming SRUB.

Based on the literature, the following treatment principles for SRUB are proposed: Intraperitoneal ruptures with large defects and high risk of complications require prompt open or laparoscopic surgical repair, as recommended by American and European urological guidelines, to prevent fatal outcomes such as intra-abdominal sepsis, infectious urinary peritonitis, or septicemia. Early diagnosis and surgery are critical for favorable prognosis. Most uncomplicated extraperitoneal ruptures tend to heal spontaneously and are managed conservatively with Foley catheter drainage and prophylactic antibiotics; spontaneous healing is expected after 2 to 3 weeks of catheterization. Delayed surgery is considered if complications arise or healing is incomplete. In confirmed intraperitoneal rupture without severe peritonitis or multi-organ injury, laparoscopic repair may be feasible. Follow-up cystography before catheter removal is essential to confirm complete healing.^[14,15]^ A systematic review of SRUB revealed that 71% of patients underwent open surgery, 13% received conservative treatment, and 4% underwent laparoscopy. The recurrence rate was significantly higher in the conservative group (23% vs 6.8%), highlighting the importance of patient education and close outpatient follow-up after discharge.^[7]^

## 4. Conclusion

SRUB should be considered in patients presenting with unexplained oliguria, ascites, and acute renal dysfunction, especially when accompanied by a history of alcohol use. Timely recognition and surgical management are vital to avoid life-threatening complications and unnecessary interventions.

## Author contributions

**Conceptualization:** Tianle Yu.

**Formal analysis:** Tianle Yu.

**Funding acquisition:** Jia Shi

**Methodology:** Tianle Yu.

**Supervision:** Chunyu Cai

**Writing – review & editing:** Tianle Yu, Yu Ji Jiang.
